# Renal, cardiac, neurological, cutaneous and coagulopathic long-term manifestations of COVID-19 after recovery; A review

**DOI:** 10.1017/S0950268822001480

**Published:** 2022-09-21

**Authors:** Reza Afrisham, Yasaman Jadidi, Maryam Davoudi, Kiana Moayedi, Saina Karami, Sahar Sadegh-Nejadi, Damoon Ashtary-Larky, ShadiSadat Seyyedebrahimi, Shaban Alizadeh

**Affiliations:** 1 Department of Clinical Laboratory Sciences, School of Allied Medicine, Tehran University of Medical Sciences, Tehran, Iran; 2 Department of Clinical Biochemistry, Faculty of Medicine, Tehran University of Medical Sciences, Tehran, Iran; 3 Student research committee, Ahvaz Jundishapur University of Medical Sciences, Ahvaz, Iran; 4 Department of Parasitology, School of Medicine, Ahvaz Jundishapur University of Medical Sciences, Ahvaz, Iran; 5 Department of Clinical laboratory, Shariati Hospital, Tehran University of Medical Sciences, Tehran, Iran; 6 Nutrition and Metabolic Diseases Research Center, Ahvaz Jundishapur University of Medical Sciences, Ahvaz, Iran; 7 Department of Hematology and Transfusion Medicine, School of Allied Medicine, Tehran University of Medical Sciences, Tehran, Iran

**Keywords:** Blood coagulation disorders, cardiac disease, COVID-19, kidney, neurological disorder, skin diseases

## Abstract

Severe acute respiratory syndrome coronavirus 2 (SARS-CoV-2) caused the novel global coronavirus disease 2019 (COVID-19) disease outbreak. Its pathogenesis is mostly located in the respiratory tract. However, other organs are also affected. Hence, realising how such a complex disturbance affects patients after recovery is crucial. Regarding the significance of control of COVID-19-related complications after recovery, the current study was designed to review the cellular and molecular mechanisms linking COVID-19 to significant long-term signs including renal and cardiac complications, cutaneous and neurological manifestations, as well as blood coagulation disorders. This virus can directly influence on the cells through Angiotensin converting enzyme 2 (ACE-2) to induce cytokine storm. Acute release of Interleukin-1 (IL1), IL6 and plasminogen activator inhibitor 1 (PAI-1) have been related to elevating risk of heart failure. Also, inflammatory cytokines like IL-8 and Tumour necrosis factor-*α* cause the secretion of von Willebrand factor (VWF) from human endothelial cells and then VWF binds to Neutrophil extracellular traps to induce thrombosis. On the other hand, the virus can damage the blood–brain barrier by increasing its permeability and subsequently enters into the central nervous system and the systemic circulation. Furthermore, SARS-induced ACE2-deficiency decreases [des-Arg9]-bradykinin (desArg9-BK) degradation in kidneys to induce inflammation, thrombotic problems, fibrosis and necrosis. Notably, the angiotensin II-angiotensin II type 1 receptor binding causes an increase in aldosterone and mineralocorticoid receptors on the surface of dendritic cells cells, leading to recalling macrophage and monocyte into inflammatory sites of skin. In conclusions, all the pathways play a key role in the pathogenesis of these disturbances. Nevertheless, more investigations are necessary to determine more pathogenetic mechanisms of the virus.

## Introduction

**Fig. 1.**
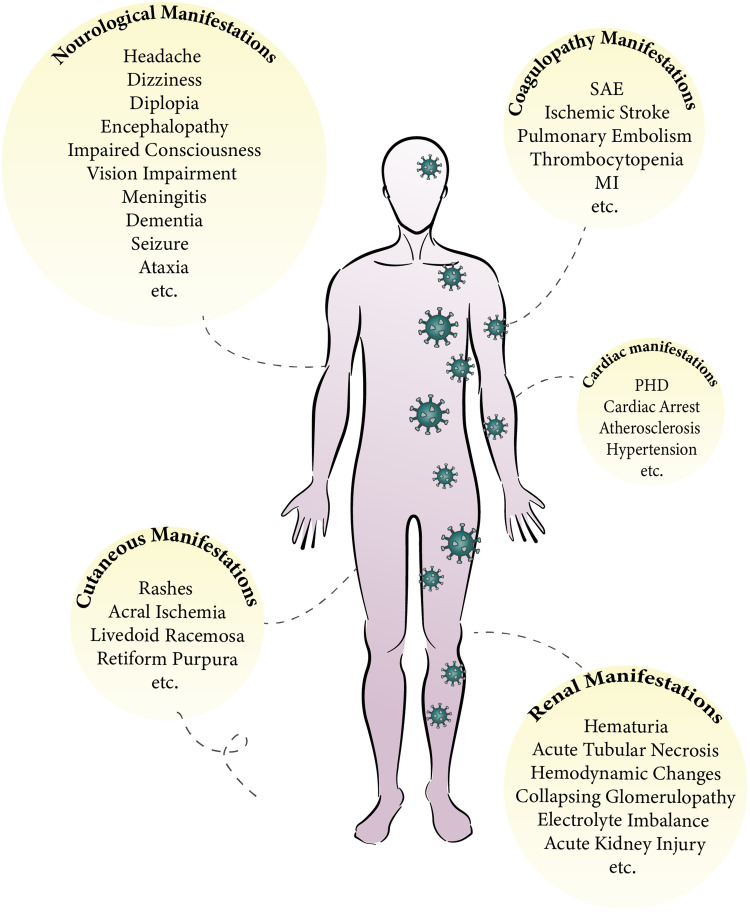
COVID-19 long-term symptoms. SAE, systemic arterial embolism; MI, myocardial infarction.

Severe acute respiratory syndrome coronavirus 2 (SARS-CoV-2) caused the novel global coronavirus disease 2019 (COVID-19) disease outbreak [1, 2]. This new virus is highly contagious as well as is a deadly virus [3]. As individuals with the virus are elevating worldwide, the complications and some disturbances caused by COVID-19 have widely increased [4]. Although, the most frequent signs of COVID-19 are respiratory symptoms [5], but, there are documents of COVID-19-correlated symptoms including blood coagulation disorders, renal and cardiac complications as well as cutaneous and neurological manifestations (Fig. 1) [3, 6–8]. As we know, Angiotensin converting enzyme 2 (ACE-2) is the receptor for coronaviruses [9] and the spike (S) protein of virus attaches it on the cell surface of some organs (e.g. cardiovascular system, kidney, lung, gastrointestinal tract (GI), skin and brain). After cleavage by transmembrane serine protease 2 (TMPRSS2), the virus enters the cell [10].

Upon viral entry, the innate immune system is recruited to fight the infection. Meanwhile, SARS-CoV-2 replicates in the pulmonary and endothelial cells of the pulmonary arteries and after destroying the cells, releases to the circulation. Macrophages and dendritic cells (DCs) endocytose the virus and present virus antigens to T helper type 1 (Th1). Further stimulation of Th1 gradually activates B cells to secrete neutralising antibodies and CD8 + T cells to destroy virus-infected cells. This stimulates the secretion of pro-inflammatory cytokines like Interlukine-6 (IL-6), IL-21, IL-18 and Tumour necrosis factor-*α* (TNF-*α*) and activates the nuclear factor kappa B (NF-*κ*B) pathway, causing a systemic hyperinflammatory state that may involve other organs [11, 12]. Along with systemic hyperinflammatory, other cellular and molecular mechanisms may influence on vital organs. So, realising how such a complex disturbance affects patients after recovery is crucial. Regarding the significance of control of COVID-19-related complications after recovery, the current review was designed to study the cellular and molecular mechanisms linking COVID-19 to significant long-term signs including renal, cardiac, cutaneous and neurological complications, as well as blood coagulation disorders. To the best our knowledge, there are no comprehensive review to explore these events related to many long-term complications of COVID-19.

## Renal manifestations

ACE2 plays a vital role in regulating the cardiovascular and renal systems via Renin-Angiotensin-Aldosterone System (RAAS). Renal juxtaglomerular cells begin to release renin in response to some conditions, including reduced renal perfusion, hypotension, ischaemia, sodium diuresis and sympathetic stimulation, which converts liver-derived angiotensinogen to angiotensin I (ANGI) [13]. Subsequently, ANGI is converted by ACE to ANGII, which in turn is further metabolised by ACE-2 into ANG I–7. Meanwhile, ANGI can also be metabolised by neutral endopeptidase to ANG 1–7. ANG1–7 and its receptor, i.e. Mas, acts as vasodilator, anti-inflammatory and anti-coagulant agents, whereas ANGII and its receptor, i.e. Angiotensin II type 1 receptor (AT1R), are vasoconstrictor, pro-inflammatory, pro-coagulant and fibrotic effectors [14]. Thus, in patients with cardiorenal diseases, ANGII can control fibrosis, hypertrophy and prooxidative hormone, which leads to an increase in salt and water retention [15, 16]. In kidneys, ACE2 is expressed on proximal tubule epithelial cells (PTECs), podocytes and endothelial cells [17]. Although the TMPRSS2 expression is low on PTECs, high levels of other proteases such as cathepsin and glutamyl aminotransferase, in these cells may help the virus entry [11]. SARS-CoV-2 moves to the kidneys through the glomerular capillaries and arteries and then enters the glomerular epithelial cells to infect the podocytes. In this way, SARS-CoV-2 enters tubular fluid and reach PTECs [13, 18]. The internalisation of ACE2 receptor induced by SARS-CoV-2 leads to ACE2 deficiency on cells, which shifts the ANG1–7-Mas pathways to angiotensin II-angiotensin II type 1 receptor (ANGII-AT1R) and induces a proinflammatory state [11].

Furthermore, ACE2 regulates the Kallikrein Kinin System. In this regards, after producing kinin from bradykininogens, this metabolite acts on both B1 and B2 receptors to induce inflammation and increase vascular permeability [14, 19, 20]. The most important agonist of B1 receptor is a bradykinin metabolite, i.e., [des-Arg9]-bradykinin (desArg9-BK), which is cleaved by ACE2. As regards SARS-induced ACE2-deficiency, it should said that not only it increases ANGII, but also decreases desArg9-BK degradation [14]. Also, Kallikrein can trigger the coagulation imbalance through activating factor 12 and plasmin. The mentioned renal changes can exacerbate systemic inflammation, thrombotic problems, fibrosis and necrosis mediated by COVID-19 in kidneys, resulting in renal damages [14].

The severity of these injuries varies from proteinuria to acute kidney injury (AKI) in ~5–70% patients [11, 14, 21]. However, a recent meta-analysis on 18 043 patients showed that 32.6% of patients admitted to the intensive care unit (ICU) suffered from AKI [22]. the incidence of AKI has been higher in non-survivors, ranging from 25–50% [23, 24]. Based on a systematic study on 193 patients with COVID-19, the corresponding figures for proteinuria, haematuria, urea nitrogen levels and creatinine levels were 44–65%, 27–44%, 14% and 10–14% [21]. Theoretically, Transient Receptor Potential Canonical Channel 6, as an ion channel in podocytes, can directly be activated by ANGII, which leads to proteinuria [13, 25]. The most common COVID-19-related renal complications are including acute tubular injury, collapsing glomerulopathy, thrombotic microangiopathy, complement activation and IL-6-induced renal vascular permeability [13, 17]. Therefore, the kidney injury leads to: (i) hypoxia-induced acute tubular necrosis with high level of creatinine, (ii) collapsing focal segmental glomerulosclerosis together with haematuria, which are common in patients with APOL1 alleles, (iii) direct viral tropism of renal tissue with high levels of micro- or macro-albuminuria and (iv) hemodynamic changes mediated by endothelitis along with electrolyte imbalance [26]. Furthermore, the infiltration of some immune cells like lymphocytes into the renal interstitium can cause cells to release more pro-inflammatory cytokines and lymphocyte-derived toxic particles (e.g. perforin), causing more kidney injuries [13]. It is worth noting that rhabdomyolysis and acute cardiorenal syndrome are also associated with kidney failure [26]. In addition, some drug-induced nephrotoxicity may also occur in COVID-19 cases; for example, remdesivir can be nephrotoxic through damaging the mitochondria in the epithelial cells of the renal tubules [27]. According to a systematic review, renovascular complications can be seen in COVID-19 cases with/without a pre-existing renal disorder. Hence, all COVID-19 patients should to be checked for their renal functions [28].

Overall, although COVID-19-related renal histopathological changes involve both parenchyma and the interstitium of kidney, glomerular injuries are milder than interstitial ones [29, 30]. These changes have already been described in a study by Faour *et al*. [13]. However, some questions still remain in this area that need further study.

## Cardiac manifestations

It has been shown that about 7.3% of COVID-19 patients suffer from heart pulsation as a primary sign [31]. The most prevalent COVID-19-related cardiovascular manifestations have been cardiac injury and myocardial injury, with 35.29% and 29.41% of cases respectively [32]. COVID-19-induced hypoxia and myocardial damage can create cardiac electrical dysregulation and arrhythmias, which is common in 30%–50% of ICU cases [33]. Moreover, about 20%–30% of hospitalised cases have had an increase in troponin I (cTnI) levels which is related to myocardial involvement [31, 34]. In cardiac tissues, ACE-2 is highly expressed in venous and arterial smooth muscle cells (SMC), endothelial cells and cardiac fibroblasts [35, 36]. It has been reported that COVID-19 can directly affect cardiomyocytes through their ACE-2 to induce inflammatory responses and cytokine storm, as aforementioned [35].

It has been offered that there is a connection between acute myocardial damages caused by COVID-19 infection and ACE-2. In fact, by shifting RAAS toward ANGII/AT1R, the level of inflammatory cytokines such as IL4, IL10 and IL6 are increased, which in turn activates T-cells in peripheral blood of COVID-19 patients. The overactivation of T cells leads to a rise in Th17 and cytotoxic CD8 T-cells [37]. In other words, when ANGII binds to AT1 receptor, atherosclerosis, hypertrophy, fibrosis, proliferation and vasoconstriction are increased, while, natriuresis and diuresis are decreased [38]. On the contrary, if ANGII binds directly to AT2 receptor or converts to ANGIII and then binds to AT2 receptor or converts to ANG (1–7) and then adjoins to ANG (1–7) receptor (MasR), atherosclerosis, hypertrophy, fibrosis, proliferation and vasoconstriction are diminished and natriuresis and diuresis are elevated [38].

The myocardial damage may occur during COVID-19-associated ‘cytokine storm’ that is followed by a Th1/Th2 imbalance and can exacerbate respiratory distress syndrome, hypoxaemia, shock and/or hypotension. Moreover, in COVID-19 patients, some factors including GCSF, IL10, IL7, IL2, IP10, MCPI, MIP1A and TNF*α* are elevated in inflammatory response [39–42]. It has been indicated that acute release of IL1, IL6 and plasminogen activator inhibitor 1 (PAI-1) were related to elevating risk of heart failure [43]. The cytokines caused the release of reactive oxygen species, superoxide anion and endogenous nitric oxide so that all of them could injure myocardial cells [44]. C-reactive protein (CRP) is an important marker in systemic inflammatory and several infections. CRP helps in stimulation of atherosclerosis and instability of atherosclerotic plaque [43]. In addition, a high prevalence of COVID-19-mediated inflammatory stress can also cause arrhythmias through inducing electrolyte and hemodynamic disorder [35, 45, 46]. In COVID-19 patients, strong interferon-mediated responses can contribute to myocardial dysfunction, especially in protective adaptive immunity by interferons [47]. It has also been recommended that severe COVID-19 patients with higher N-terminal pro-brain natriuretic peptide (NT-proBNP) levels were older patients who had high levels of markers of systemic inflammatory. NT-proBNP levels presented as an independent risk factor of in-hospital survival rate in severe COVID-19 patients. So that, COVID-19 patients with higher NT-proBNP (above 88.64 pg/ml) levels had a lower survival rate [48].

Transforming growth factor-*β*1 (TGF-*β*1) acts as important mediator in the process of tissue fibrosis and leads to scarring by activating its downstream small mother against decapentaplegic (Smad) pathway [49]. In this direction, SARS-CoV-2 activates TGF-*β* signalling by the smad pathway to stimulating lung fibrosis. In addition, smad pathway activated by COVID-19 is a prevalent pathway of interstitial fibrosis development in the myocardium [47]. In fact, TGF-*β* signalling in COVID-19 process is activated by both the non-canonical and the canonical signalling pathways [50]. A papain-like protein as well as a nucleocapsid protein along with smad3 activate the canonical and the non-canonical pathways, so, TGF-*β* is upregulated [50].

Overall, various cellular and molecular mechanisms such as recognition of ACE-2 and receptor infection, cytokine storm and induction in several inflammatory responses are contributed in cardiac dysfunction, which can increase pulmonary vascular resistance, as well as result in pulmonary heart disease and hypertension.

## Neurological complications

Besides the acute clinical manifestation of respiratory virus, understanding the neurological complications following COVID-19 illness will be crucial [51]. A tremendous number of clinical studies have reported that many of COVID-19 patients had at least one neurological manifestation [7]. In a 2022 meta-analysis, 2791 out of 18 258 COVID-19 patients had neurological symptoms with the mortality rate of 29.1% [52]. A scoping review on neurological manifestations in COVID-19 patients has revealed that the spectrum of these symptoms ranged from mild to severe [53]. The mild manifestations included olfactory and gustatory disorders, dizziness, headache, vomiting, malaise, fatigue and anorexia. Whereas, ischaemic stroke, impaired consciousness, encephalopathy, cognition and memory impairments, meningitis, diplopia and ophthalmoplegia are regarded as severe symptoms [23, 54, 55].

Neurologic complications can also be divided into central nervous system (CNS) and peripheral nervous system (PNS) involvements. The CNS involvement comprises headache and dizziness, impaired consciousness, acute cerebrovascular disease, corticospinal tract signs, ataxia and seizure, meningitis, encephalitis and stroke. While, the PNS involvement includes hyposmia and dysgeusia, vision impairment, nerve pain and Guillian-Barre syndrome [56, 57].

Interestingly, sex and age differences have been reported to affect the severity of neurological manifestations. Differences in humoral and innate immune responses to viral infections between men and women as well as age-related expression of ACE2 are supposed to be involved [58, 59]. In addition, Heart disorders, diabetes and dyslipidemia have increased the risk of developing neurological complications in COVID-19 by 2-fold in a meta-analysis on 4401 patients [60]. [59] Moreover, some neurological symptoms like Parkinsonism can be developed in cases who recovered from COVID-19 as shown on a clinical study. Thus, Parkinsonism is a post-COVID neurological symptom [61].

**Fig. 2.**
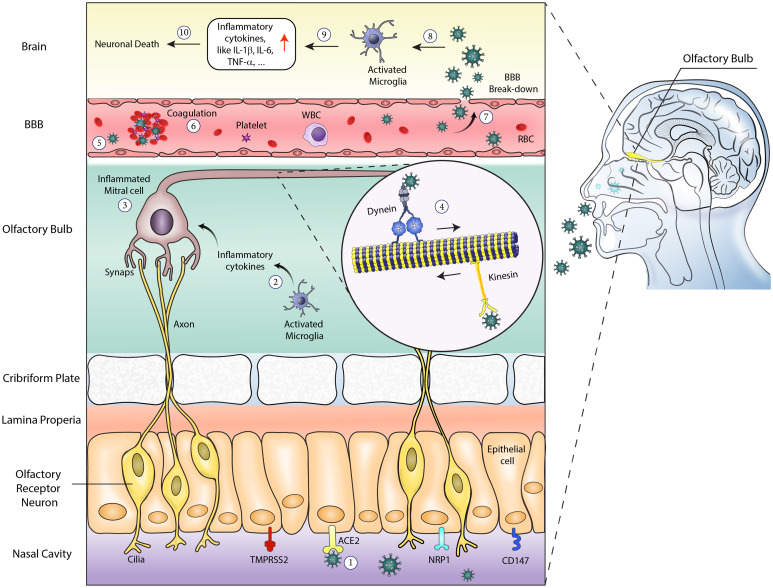
Post-COVID 19 neurological disorders. (1) viral entrance through ACE2 receptor; (2) inflammatory cytokines increase due to activated microglia; (3) SARS-Cov 2 leads to inflammated mitral cells; (4) virus migration through motor proteins such as dynein and kinesin; (5) virus BBB infiltration; (6) virus may cause coagulation; (7) BBB break-down serve as a gate for virus penetration into the brain; (8) virus activates microglial cells; (9) rise in inflammatory cytokines; (10) neuronal death occurrence. TMPRSS2, Transmembrane protease, serine 2; ACE2, Angiotensin-converting enzyme 2; NRP1, neuropilin-1; CD147, cluster of differentiation 147; BBB, blood–brain barrier; WBC, White blood cells; RBC, Red blood cells.

However, the aetiology for COVID-19 neurological consequences is not completely understood. Post- COVID 19 neurological disorders are shown in Figure 2. From the existent studies on the neuropathogenicity of COVID-19, two possible underlying mechanisms have been proposed: (1) Direct mechanisms and (2) Indirect mechanisms [7, 53]. In direct invasion, SARS-CoV-2 could affect the nervous system mainly through the neuronal pathways and to a lesser extent through the blood circulation pathways or lymphatic system [23, 54]. In terms of neuronal pathway, the virus mainly spreads via the transcribial route from the olfactory epithelium along with the olfactory nerve to the olfactory bulb in the nasal cavity and forebrain [62]. This latter route effectively makes it a channel among the nasal epithelium, the entire brain and cerebrospinal fluid and subsequently causes inflammation and demyelinating reactions [58, 59, 62, 63]. Another potential mechanism is the neuronal retrograde or anterograde transmission. In this route, the viruses invade the sensory or motor nerve endings and migrate by the motor proteins including dynein and kinesins [64]. These mentioned pathways can lead to dysfunction of the respiratory and/or gastrointestinal centres [65].

Accumulating evidence suggests that SARS-CoV-2 gains entry to the CNS through the ACE2 and TMPRSS2 receptors on sustentacular cells in the human olfactory neuroepithelium and several brain structures as well as via CD147 and Neuropilin-1 (NRP1) receptors in sensory neuron [7, 23, 51, 53–59, 62–66]. Based on studies, both ACE2 and NRP1 are highly expressed in the brain. Although ACE2 is mostly expressed in circumventricular organs and endothelial vasculature, NRP1 is generally expressed in the hippocampus, endothelial cells, mural cells, perivascular macrophages and microglia [67, 68]. Following the dissemination of the coronaviruses in the nervous system, it can damage the blood–brain barrier by increasing its permeability and subsequently enter into the CNS and the systemic circulation [56, 57].

In addition to direct infection injury, the nervous system may be affected indirectly due to a severe systemic reaction in response to a viral infection outside the nervous system. In this regard, neuro-inflammation, hypoxia, coagulation disorders, dysregulated blood pressure (due to binding to ACE2 receptors) as well as altered glucose and lipid metabolism are considered as indirect possible mechanisms in neurological consequences of COVID-19 [23, 53–59, 62–65].

It has been supposed that the neurotropic potential of the COVID-19 is closely related to the development of a systemic inflammatory response syndrome (SIRS) [58]. SIRS mediates the release of inflammatory cytokines (cytokine storm) and free radicals, which affect the central and peripheral nervous system. As a consequence of a cytokine storm [cytokines (e.g., IL-6, IL-1TNF-*α*, IL-1*β*, IFN-*γ*, IL-4, IL-10)], glial cells will be activated [53, 54]. This activation can induce a pro-inflammatory state which is positively correlated with the severity of neurological symptoms [69]. Encephalitis and encephalopathy have been related to a rise in the levels of proinflammatory cytokines and antioxidants based on two clinical studies [70, 71]. On the other hand, headache has less been associated with mortality since patients with headaches have experienced less cellular effectors related to cytokine storm, including D-dimer and ferritin [72].

Another detrimental effect of coronaviruses is hypoxia injury that caused by lung tissue lesions. This, in turn, leads to subsequent nervous system damage such as interstitial oedema and encephalopathy. Moreover, headache due to ischaemia, congestion and even coma can be observed [7]. Interestingly, hypoxia has been implicated in the tau hyperphosphorylation in Alzheimer's. Furthermore, increased ACE expression in brain of Alzheimer's patients suggests that COVID-19 may contribute the incidence of this neurodegenerative disease as well [73, 74].

A review of current published literatures has indicated that coagulation dysfunction was another indirect mechanism in case of neuroinvasive potential of SARS-CoV2. Coagulopathy, including elevated D-dimer levels, prolonged prothrombin time, decreased platelet counts, elevated PAI-1 and Von Willebrand factor (VWF) have been highlighted in severe cases [23, 53–59, 62–65]. It may render these patients prone to ischaemic strokes and other cerebrovascular events [54].

To put these findings together, understanding the neurotropic characteristics of SARS-CoV-2 is substantial to individualise and prioritise the treatment protocols in the context of patients with COVID-19. However, more studies are needed to clarify the underlying mechanism of neuropathogenicity of the new coming virus.

## Cutaneous manifestations

Cutaneous manifestations of COVID-19 are heterogeneous and occur in 1–20% of cases. Many studies classify them into five general categories, which are summarised in Table 1 [75, 76]. Overall, the spike protein of SARS-CoV-2 enters the skin through ACE2 receptors on the surface of epithelial cells, keratinocytes, endothelial vessels and eccrine glands that can directly damage them [77]. Upon virus entry, innate immunity is activated to prevent viral replication and plasmacytoid dendritic cells (pDCs) establish synapse with infected cells and upregulate toll-like receptor 7 (TLR7) and release the type-I interferons (IFNI) [78, 79]. Indeed, TLRs sense pathogen-associated molecular patterns (PAMPs) such as single-stranded RNA, unmethylated double-stranded DNA (CpG), lipoproteins, flagellin and lipopolysaccharide that induce the secretion of inflammatory cytokines [80]. In chronic infections and autoimmune diseases, pDCs are constantly activated and participate in the pathogenesis through IFNI overactivity [81]. Thus, the most skin lesions, including erythematous/vesicular /urticarial rashes, are those that are also common in other viral infections (e.g. *Herpesviridae*). Additionally, some legions may either be exacerbated by co-infection/reactivation of SARS-CoV-2 with other viruses or by a drug-induced reaction; however, a combination of the two is also possible [75, 76, 82].

**Table 1.** The classification of common skin lesions based on their target groups, frequency, COVID-19 severity, timing and mechanisms

Groups	Subjects	Frequency among COVID-19-associated cutaneous manifestations	COVID-19 severity	Timing of onset and persistence	Possible mechanisms
Erythematous rashes (e.g. maculopapular and pityrriasis-like rosea)	Adults	50%	Severe	Simultaneously with other symptoms 7−9 days	Common in viral infections Type IV DHR Co-infection/reactivation
Vesicular rashes	Adults	15%	Moderate	After other symptoms 10−15 days	Co-infection/reactivation Common in viral infections
Urticarial rashes	Adults/ youngers	9–19%	Severe	Before other symptoms (as an early sign) Simultaneously or a few weeks after other symptoms 6−8 days	Common in viral upper respiratory tract infections Complement Type I DHR (IgE mediated) Co-infection/reactivation
Vascular origin					
Chilblain-like	Youngers	common	Mild and asymptomatic	Late-onset 12–14 days	IFNI Vasospasm Autoantibodies
Ischaemic lesions	Elderly	Rare	Critically ill	Late-onset in drug-induced types Simultaneously with other symptoms in viral-induced types	Thrombosis and complement deposition Type III DHR

DHR, drug hypersensitivity reaction; IgE, immunoglobulin E.

The other two types of eruptions are of vascular origin: (1) chilblain- or pernio-like and (2) ischaemic types such as livedoid racemosa, retiform purpura and acral ischaemia. A histopathological study shows the perivascular and perieccrine infiltration of T lymphocytes and pDCs in these legions [78]. Chilblain-like is a late-onset eruption, thus PCR may be negative, that may exhibit in young people with mild and asymptomatic COVID-19 through IFNI responses and an endothelial dysfunction. Since youngers have stronger innate immune systems; they can inhibit the virus without activating acquired immunity, resulting in negative antibody tests [83]. Nevertheless, the ischaemic legions are related to hypercoagulability and complement deposition in elderly with severe COVID-19. In critical COVID-19, the impairment of IFNI responses downregulates ACE2, shifting the RAAS toward ANGII. ANGII in turn stimulates T cells to increase the expression of AT1R [78, 84]. The ANGII-AT1R binding causes an increase in aldosterone and mineralocorticoid receptors on the surface of DC cells, leading to the production of pro-inflammatory cytokines (such as IL-6) and further recalling macrophage and monocyte into the inflammatory sites of skin [78]. Furthermore, in ischaemic lesions of patients with pulmonary thromboembolism, complement induces thrombotic microvascular injury through mannan binding lectin (MBL) pathways [85, 86]. In other words, the viral spike glycoproteins enter directly into ACE2-containing cells and through their glycan moiety interact with MBL, activating it to increase IL6, followed by platelet aggregation and complement deposition [85]. In Chilblain, however, an increase in IFN1 upregulates ACE2 and its vasoprotective mechanism via shifting the RAAS toward ANG1–7, resulting in only an impermanent acral vasospasm [78, 83]. Both vasospasm and thrombotic states can also mediate a local hypoxia in the cutaneous vasculature, which inhibit the regulatory T cells and their anti-inflammatory checkpoints by stimulating Hypoxia-inducible factor 1-alpha and induce a cutaneous endothelial dysfunction [87, 88]. Ethnic, age, gender, hormonal and genetic factors can also affect the skin pathogenesis of COVID-19 by interfering with any of these pathways [78, 83].

Moreover, the humoral immunity dysregulation induced by COVID-19 can produce autoantibodies (including antiphospholipid antibodies), which may cause skin lesions through inducing an autoimmune reaction mediated by molecular mimicry, immune complex-trigged inflammation and prothrombotic state [83]. In one study, the injection of purified IgG from COVID-19 cases into thrombotic mice could accelerate thrombus formation [89]. This is probably the mechanism by which multisystem inflammatory syndrome develops cutaneous and organ damages in children after 4–6 weeks of COVID-19. More importantly, the activation of antibody-secreting cells needs several weeks, thus autoantibody-mediated lesions usually are a late-onset outcome [90].

Overall, the reviewed studies show that there is a link between skin manifestations and COVID-19, but whether SARS-CoV-2 is the cause of other underlying conditions is still unclear and needs further investigation.

## Coagulopathic disorders

COVID-19 patients are also at emerging risk of thrombosis disorder. Two meta-analysis studies showed that the rates of the deep vein thrombosis, pulmonary embolism and arterial thrombus were 19.8%, 18.9% and 3.7%, respectively [91, 92]. Moreover, thrombogenesis was highly associated with the mortality so that 50% of nonsurvivors have shown a procoagulant state, while only 7% of survivors have presented this symptom [93]. Strikingly, thrombosis is a part of the innate immune reaction to pathogens that is called immunothrombosis [94, 95]. Neutrophil activation with neutrophil extracellular traps (NETOSIS), endothelial cell lesion and activation, as well as platelet activation and aggregation, altogether with coagulation protease activation, take a part in the process of immunothrombosis, particularly in lung [94, 96]. In addition, PAMPs, damage-associated molecular patterns (DAMPs), along with extracellular histones and DNA, have recently been considered in immunothrombosis. PAMPs and DAMPs raise TF explanation on monocytes and stimulate the process of Neutrophil extracellular traps (NETs) so that, immunothrombosis is induced [94, 97, 98]. Cell-free DNA and extracellular histones are the important parts of NETs which are secreted from dead cells, and they increase the inflammation and induce the thrombosis via damaging fibrinolysis, raising thrombin production and stimulating platelet activation [94, 98, 99]. Based on clinical studies, the severity of COVID-19 is a key factor to increase the level of NETs in blood, tracheal aspirates and different organs of patients [100–102].

It has been offered that high fibrin degradation product levels (called D-dimer), long prothrombin time (PT), activated partial thromboplastin times, reduction in platelet counts, raised levels of fibrinogen, are commonly occurred in COVID-19 patients during thrombosis [94]. A considerable elevation of factors v, viii; and VWF has also been indicated in intense COVID-19 patients. Furthermore, high plasma levels of VWF antigen and pro-peptide are revealed in severe COVID-19 which they are indicative of endothelial stimulation as well as injury by secretion of VWF from Weibel-Plade bodies [94, 103]. In a study in 2020, Goshua *et al*. evaluated COVID-19 associated coagulopathy. They indicated that P_selectin and VWF antigen (markers of endothelial cell and platelet activation), as well as soluble thrombomodulin (a marker of endothelial cell activation) were significantly increased in ICU patients in comparison with non-ICU patients. In addition, they reported mortality to be significantly related to soluble thrombomodulin and VWF antigen among all patients [103].

Platelets release VWF cumulative in *α*-granules just till activation. ADAMTS-13 (a disintegrin and metalloproteinase with a thrombospondin type 1 motif, member 13) is expressed by hepatic stellate and endothelial cells [104, 105]. ADAMTS-13 controls the activation of VWF via excision of ultra-large VWF multimers (> 10 000 KDa) which are released from endothelial cells into active high molecular weight multimers (< 10 000 KDa) under stress states [106]. Intense deficiency of ADAMTS-13 results in accumulation of ultra-large VWF multimers and causes microvascular thrombosis, thrombocytopenia and thrombotic thrombocytopenic purpura (TTP) [107]. A mechanism that notably contributes in ADAMTS-13 deficiency is linked to the antiphospholipid antibody production within SARS-CoV-2 infection [108–111]. Antiphospholipid antibodies have been incompatibility announced in all patients of COVID-19 [108, 110, 111]. Despite, the prolongation of aPTT [111], the patients with antiphospholipid syndrome have unusual ADAMTS-13 plasmatic activity, so that, an increased risk of thrombosis is seen [112]. Antiphospholipid antibodies during active SARS-CoV-2 infection maybe attach the spacer domain of ADAMTS-13 which can intervene in the identification and proteolysis of VWF [113]. In this regard, studies reported that COVID-19 nonsurvivors have shown higher VWF and lower ADAMTS13 compared with survivors [114, 115].

Overall it can be seen that TLRs-3 and −7 are activated by the viral RNA to induce the NF-*κ*B Pathway and the interferon regulatory factors, that they raise the production and release of pro-inflammatory cytokines [94]. This pro-inflammatory condition has been seen in several viral diseases such as influenza and SARS-CoV-2 [113]. The elevation of these pro-inflammatory cytokines such as IL-1, IL-6 and TNF*α* can promote thrombosis [103]. So that, inflammatory cytokines (IL-8 and TNF- *α*) cause the secretion of VWF from human endothelial cells and then VWF binds to NETs to induce thrombosis [113]. Moreover, proinflammatory situation is influential on haemostasis by blocking of fibrinolysis [113]. Also increased levels of myeloperoxidase as a NET-linked marker have been demonstrated in COVID-19 patients [94]. More importantly, the interaction among complement activation, inflammation and the coagulation cascade is seen to be crucial to understanding the COVID-19 pathophysiology and is involved to triggering disseminated intravascular coagulation [116]. Membrane attack complex (C5b-9) and C3a are both responsible for platelet activation. Moreover, C5a increases cellular and plasma TF expression [117]. Moreover, it has been established that considerable hypoxaemia in COVID-19 patients is caused in prothrombotic conditions through stimulation of endothelial synthesis of procoagulants, upregulation of plasminogen activator inhibitor and releasing tissue factor and VWF as well [113].

On the other hand, a large number of patients with non-valvular atrial fibrillation who use direct oral anticoagulants (DOACs) (to prevent systemic embolism and stroke) may be treated with antiviral drugs (lupinavid/rynovid, varonavid), cloquine or hydroxychloroquine, antibiotics and tocilizumab for the treatment of severe respiratory syndrome caused by COVID-19 [118]. Since both DOACs and antiviral drugs are substrates of cytochrome P450-based metabolic pathways; the simultaneous use of these two drugs can greatly elevate the plasma level of DOACs and lead to an increased risk of uncontrolled bleeding and thrombotic complications [118, 119].

In conclusion, hypercoagulability, as a noticeable feature of COVID-19, can cause thrombotic vascular events. Several mechanisms may be involved in the occurrence of the thrombosis, such as inflammatory storm, renin angiotensin system dysregulation and uncontrolled inflammation-mediated endothelial injury. However, to get a better understanding of COVID-19, more studies are needed.

## Conclusions

Regarding the significance of control of COVID-19-related complications after recovery, the current study has summarised to review the cellular and molecular mechanisms linking COVID-19 to some long-term symptoms including renal, cardiac, cutaneous, neurological and coagulopathic complications. As mentioned earlier, this virus can directly influence on the cells through ACE-2 to induce cytokine storm to increase the risk of heart failure and thrombosis. On the other hand, the virus can damage the blood–brain barrier by increasing its permeability and subsequently enters into the CNS and the systemic circulation. Furthermore, SARS-induced ACE2-deficiency decreases desArg9-BK degradation in kidneys to induce inflammation, thrombotic problems, fibrosis and necrosis. Notably, the ANGII-AT1R binding causes an increase in aldosterone and mineralocorticoid receptors on the surface of DC cells, leading to recalling macrophage and monocyte into inflammatory sites of skin. All the pathways play a key role in the pathogenesis of these disturbances and physicians should be aware in their attention to all these complications and take precision steps for control, screening and even cure. Nevertheless, more investigations are necessary to determine more pathogenetic mechanisms of the virus.

## Data Availability

Not applicable.

## References

[1] Mitsuyama K (2020) Clinical features and pathogenic mechanisms of gastrointestinal injury in COVID-19. Journal of Clinical Medicine 9, 3630. 33187280 10.3390/jcm9113630 PMC7696882

[2] Mandal A (2020) Gastrointestinal manifestations in COVID-19 infection and its practical applications. Cureus 12, 8750. 10.7759/cureus.8750 PMC7377012 32714688

[3] Nami M (2020) The interrelation of neurological and psychological symptoms of COVID-19: risks and remedies. Journal of Clinical Medicine 9, 2624. 32823540 10.3390/jcm9082624 PMC7464612

[4] van Vuren EJ (2021) The neuropsychiatric manifestations of COVID-19: interactions with psychiatric illness and pharmacological treatment. Biomedicine & Pharmacotherapy 135, 111200. 33421734 10.1016/j.biopha.2020.111200 PMC7834135

[5] Bodnar B (2021) Cellular mechanisms underlying neurological/neuropsychiatric manifestations of COVID-19. Journal of Medical Virology 93, 1983–1998. 33300152 10.1002/jmv.26720 PMC7897247

[6] Mihalopoulos M (2020) COVID-19 and kidney disease: molecular determinants and clinical implications in renal cancer. European Urology Focus 6, 1086–1096. 32540268 10.1016/j.euf.2020.06.002 PMC7280142

[7] To KK-W (2020) Consistent detection of 2019 novel coronavirus in saliva. Clinical Infectious Diseases 71, 841–843. 32047895 10.1093/cid/ciaa149 PMC7108139

[8] Lopez-Leon S (2021) More than 50 long-term effects of COVID-19: a systematic review and meta-analysis. Scientific reports 11, 1–12. 34373540 10.1038/s41598-021-95565-8 PMC8352980

[9] Hoffmann M (2020) SARS-CoV-2 cell entry depends on ACE2 and TMPRSS2 and is blocked by a clinically proven protease inhibitor. Cell 181, 271–280. 32142651 10.1016/j.cell.2020.02.052 PMC7102627

[10] Singh H (2021) ACE2 and TMPRSS2 polymorphisms in various diseases with special reference to its impact on COVID-19 disease. Microbial Pathogenesis 150, 104621. 33278516 10.1016/j.micpath.2020.104621 PMC7709597

[11] Ahmadian E (2021) COVID-19 and kidney injury: pathophysiology and molecular mechanisms. Reviews in Medical Virology 31, 2176. 10.1002/rmv.2176 PMC7646060 33022818

[12] Silva MJA (2022) Innate immunity to SARS-CoV-2 infection: a review. Epidemiology & Infection 150, 1–49. 10.1017/S095026882200125X PMC9354479 35843719

[13] Faour WH (2021) Mechanisms of COVID-19-induced kidney injury and current pharmacotherapies. Inflammation Research 71, 1–18. 10.1007/s00011-021-01520-8 PMC8606168 34802072

[14] de Carvalho PR, Sirois P and Fernandes PD (2021) The role of kallikrein-kinin and renin-angiotensin systems in COVID-19 infection. Peptides 135, 170428. 33065209 10.1016/j.peptides.2020.170428 PMC7553876

[15] Quadri SS (2016) Interaction of the renin angiotensin and Cox systems in the kidney. Frontiers in Bioscience (Scholar Edition) 8, 215. 27100703 10.2741/s459 PMC5119519

[16] Culver S, Li C and Siragy HM (2017) Intrarenal angiotensin-converting enzyme: the old and the new. Current Hypertension Reports 19, 1–7. 28929450 10.1007/s11906-017-0778-2 PMC5913745

[17] Chen A (2021) Similarities and differences between COVID-19-associated nephropathy and HIV-associated nephropathy. Kidney Diseases 8, 1–12. 35127839 10.1159/000520235 PMC8805054

[18] Ye M (2006) Glomerular localization and expression of angiotensin-converting enzyme 2 and angiotensin-converting enzyme*:* implications for albuminuria in diabetes. Journal of the American Society of Nephrology 17, 3067–3075. 17021266 10.1681/ASN.2006050423

[19] Marceau F (1995) Kinin B1 receptors: a review. Immunopharmacology 30, 1–26. 7591710 10.1016/0162-3109(95)00011-h

[20] Donoghue M (2000) A novel angiotensin-converting enzyme–related carboxypeptidase (ACE2) converts angiotensin I to angiotensin 1–9. Circulation Research 87, e1–e9. 10969042 10.1161/01.res.87.5.e1

[21] Vahdat S (2022) Clinical profile, outcome and management of kidney disease in COVID-19 patients–a narrative review. European Review for Medical and Pharmacological Sciences 26, 2188–2195. 35363369 10.26355/eurrev_202203_28367

[22] Passoni R (2021) Occurrence of acute kidney injury in adult patients hospitalized with COVID-19: a systematic review and meta-analysis. Nefrología 42, 404–414. 36460430 10.1016/j.nefroe.2022.11.005 PMC9707651

[23] Chen T (2020) Clinical characteristics of 113 deceased patients with coronavirus disease 2019: retrospective study. Bmj 368, 1091. 10.1136/bmj.m1091 PMC7190011 32217556

[24] Zhou F (2020) Clinical course and risk factors for mortality of adult inpatients with COVID-19 in Wuhan, China: a retrospective cohort study. The Lancet 395, 1054–1062. 10.1016/S0140-6736(20)30566-3 PMC7270627 32171076

[25] Hoffmann S (2004) Angiotensin II type 1 receptor overexpression in podocytes induces glomerulosclerosis in transgenic rats. Journal of the American Society of Nephrology 15, 1475–1487. 15153558 10.1097/01.asn.0000127988.42710.a7

[26] Liakopoulos V (2021) COVID-19 and the kidney: time to take a closer look. International Urology and Nephrology 54, 1–5. 34383205 10.1007/s11255-021-02976-7 PMC8358250

[27] Legrand M (2021) Pathophysiology of COVID-19-associated acute kidney injury. Nature Reviews Nephrology 17, 751–764. 34226718 10.1038/s41581-021-00452-0 PMC8256398

[28] Kaur A (2022) Renovascular complications in Covid19: a systematic review. International Journal of Early Childhood 14, 2022.

[29] Diao B (2021) Human kidney is a target for novel severe acute respiratory syndrome coronavirus 2 infection. Nature Communications 12, 1–9. 10.1038/s41467-021-22781-1 PMC8096808 33947851

[30] Ferlicot S (2021) The spectrum of kidney biopsies in hospitalized patients with COVID-19, acute kidney injury and/or proteinuria. Nephrology Dialysis Transplantation 36, 1253–1262. 10.1093/ndt/gfab042 PMC7928708 33576823

[31] Welsh P (2019) Cardiac troponin T and troponin I in the general population: comparing and contrasting their genetic determinants and associations with outcomes. Circulation 139, 2754–2764. 31014085 10.1161/CIRCULATIONAHA.118.038529 PMC6571179

[32] Pratistha FSM (2022) Systematic review of cardiovascular manifestations in COVID-19 and management consideration. Open Access Macedonian Journal of Medical Sciences 10, 332–339.

[33] Pranata R, Huang I and Raharjo SB (2020) Incidence and impact of cardiac arrhythmias in coronavirus disease 2019 (COVID-19): a systematic review and meta-analysis. Indian Pacing and Electrophysiology Journal 20, 193–198. 32814094 10.1016/j.ipej.2020.08.001 PMC7428753

[34] Mitrani RD, Dabas N and Goldberger JJ (2020) COVID-19 cardiac injury: implications for long-term surveillance and outcomes in survivors. Heart Rhythm 17, 1984–1990. 32599178 10.1016/j.hrthm.2020.06.026 PMC7319645

[35] Khan I (2020) At the heart of COVID-19 [published online ahead of print May 5, 2020]. Journal of Cardiac Surgery 72, 799–404.

[36] Guo J (2020) Coronavirus disease 2019 (COVID-19) and cardiovascular disease: a viewpoint on the potential influence of angiotensin-converting enzyme inhibitors/angiotensin receptor blockers on onset and severity of severe acute respiratory syndrome coronavirus 2 infection. Journal of the American Heart Association 9, e016219. 32233755 10.1161/JAHA.120.016219 PMC7428639

[37] Xu Z (2020) Pathological findings of COVID-19 associated with acute respiratory distress syndrome. The Lancet Respiratory Medicine 8, 420–422. 32085846 10.1016/S2213-2600(20)30076-X PMC7164771

[38] Tajbakhsh A (2021) COVID-19 and cardiac injury: clinical manifestations, biomarkers, mechanisms, diagnosis, treatment, and follow up. Expert Review of Anti-infective Therapy 19, 345–357. 32921216 10.1080/14787210.2020.1822737

[39] Zheng Y-Y (2020) COVID-19 and the cardiovascular system. Nature Reviews Cardiology 17, 259–260. 10.1038/s41569-020-0360-5 PMC7095524 32139904

[40] Huang C (2020) Clinical features of patients infected with 2019 novel coronavirus in Wuhan, China. The Lancet 395, 497–506. 10.1016/S0140-6736(20)30183-5 PMC7159299 31986264

[41] Wei Z and Qian H (2020) Myocardial injury in patients with COVID-19 pneumonia. Zhonghua xin xue guan bing za zhi 48, E006. 32118393 10.3760/cma.j.issn.cn112148-20200220-00106

[42] Wong C (2004) Plasma inflammatory cytokines and chemokines in severe acute respiratory syndrome. Clinical & Experimental Immunology 136, 95–103. 15030519 10.1111/j.1365-2249.2004.02415.x PMC1808997

[43] Wang J (2020) Dysfunctional coagulation in COVID-19: from cell to bedside. Advances in Therapy 37, 3033–3039. 32504450 10.1007/s12325-020-01399-7 PMC7274265

[44] Sattar Y (2020) COVID-19 cardiovascular epidemiology, cellular pathogenesis, clinical manifestations and management. IJC Heart & Vasculature 29, 100589. 32724831 10.1016/j.ijcha.2020.100589 PMC7359794

[45] Bhatla A (2020) COVID-19 and cardiac arrhythmias. Heart Rhythm 17, 1439–1444. 32585191 10.1016/j.hrthm.2020.06.016 PMC7307518

[46] Kochi AN (2020) Cardiac and arrhythmic complications in patients with COVID-19. Journal of Cardiovascular Electrophysiology 31, 1003–1008. 32270559 10.1111/jce.14479 PMC7262150

[47] Babapoor-Farrokhran S (2020) Myocardial injury and COVID-19: possible mechanisms. Life Sciences 253, 117723. 32360126 10.1016/j.lfs.2020.117723 PMC7194533

[48] Gao L (2020) Prognostic value of NT-proBNP in patients with severe COVID-19. Respiratory Research 21, 1–7. 32293449 10.1186/s12931-020-01352-w PMC7156898

[49] Hu H-H (2018) New insights into TGF-*β*/Smad signaling in tissue fibrosis. Chemico-Biological interactions 292, 76–83. 30017632 10.1016/j.cbi.2018.07.008

[50] Carlson Jr FR (2020) Multiorgan damage in patients with COVID-19: is the TGF-*β*/BMP pathway the missing link? Basic to Translational Science 5, 1145–1148. 32984657 10.1016/j.jacbts.2020.09.003 PMC7508496

[51] Peterson CJ, Sarangi A and Bangash F (2021) Neurological sequelae of COVID-19: a review. *The Egyptian Journal of Neurology*. Psychiatry and Neurosurgery 57, 1–8. 10.1186/s41983-021-00379-0 PMC8424148 34511868

[52] Mahdizade Ari M (2022) Neurological manifestations in patients with COVID-19: a systematic review and meta-analysis. Journal of Clinical Laboratory Analysis 36, e24403. 35385200 10.1002/jcla.24403 PMC9102520

[53] Chen X (2021) A systematic review of neurological symptoms and complications of COVID-19. Journal of Neurology 268, 392–402. 32691236 10.1007/s00415-020-10067-3 PMC7370630

[54] Wenting A (2020) COVID-19 neurological manifestations and underlying mechanisms: a scoping review. Frontiers in Psychiatry 11, 860. 32973590 10.3389/fpsyt.2020.00860 PMC7472775

[55] Lechien JR (2020) Olfactory and gustatory dysfunctions as a clinical presentation of mild-to-moderate forms of the coronavirus disease (COVID-19): a multicenter European study. European Archives of Oto-Rhino-Laryngology 277, 2251–2261. 32253535 10.1007/s00405-020-05965-1 PMC7134551

[56] Baig AM (2020) Evidence of the COVID-19 virus targeting the CNS: tissue distribution, host–virus interaction, and proposed neurotropic mechanisms. ACS Chemical Neuroscience 11, 995–998. 32167747 10.1021/acschemneuro.0c00122

[57] Tandon M (2021) A comprehensive systematic review of CSF analysis that defines neurological manifestations of COVID-19. International Journal of Infectious Diseases 104, 390–397. 33434662 10.1016/j.ijid.2021.01.002 PMC7837002

[58] Liguori C (2020) Subjective neurological symptoms frequently occur in patients with SARS-CoV2 infection. Brain, Behavior, and Immunity 88, 11–16. 32416289 10.1016/j.bbi.2020.05.037 PMC7235586

[59] Sullivan BN and Fischer T (2021) Age-associated neurological complications of COVID-19: a systematic review and meta-analysis. Frontiers in Aging Neuroscience 13, 374. 10.3389/fnagi.2021.653694 PMC8366271 34408638

[60] Radwan N (2022) Neurological associations Among COVID-19 patients: a systematic review and meta-analysis. Dr Sulaiman Al Habib Medical Journal 4, 1–11.

[61] Ali SS (2022) New-onset Parkinsonism as a COVID-19 infection sequela: a systematic review and meta-analysis. Annals of Medicine and Surgery 80, 104281. 35971509 10.1016/j.amsu.2022.104281 PMC9359766

[62] Reza-Zaldívar EE (2021) Infection mechanism of SARS-COV-2 and its implication on the nervous system. Frontiers in Immunology 11, 3738. 10.3389/fimmu.2020.621735 PMC7878381 33584720

[63] Desforges M (2020) Human coronaviruses and other respiratory viruses: underestimated opportunistic pathogens of the central nervous system? Viruses 12, 14. 10.3390/v12010014 PMC7020001 31861926

[64] Guillaud L (2020) Anterograde axonal transport in neuronal homeostasis and disease. Frontiers in Molecular Neuroscience 13, 179. 10.3389/fnmol.2020.556175 PMC7531239 33071754

[65] Whittaker A, Anson M and Harky A (2020) Neurological manifestations of COVID-19: a systematic review and current update. Acta Neurologica Scandinavica 142, 14–22. 32412088 10.1111/ane.13266 PMC7273036

[66] Fodoulian L (2020) SARS-CoV-2 receptors and entry genes are expressed in the human olfactory neuroepithelium and brain. IScience 23, 101839. 33251489 10.1016/j.isci.2020.101839 PMC7685946

[67] Hernández VS (2021) ACE2 Expression in rat brain: implications for COVID-19 associated neurological manifestations. Experimental Neurology 345, 113837. 34400158 10.1016/j.expneurol.2021.113837 PMC8361001

[68] Davies J (2020) Neuropilin-1 as a new potential SARS-CoV-2 infection mediator implicated in the neurologic features and central nervous system involvement of COVID-19. Molecular Medicine Reports 22, 4221–4226. 33000221 10.3892/mmr.2020.11510 PMC7533503

[69] Almutairi MM (2021) Neuroinflammation and its impact on the pathogenesis of COVID-19. Frontiers in Medicine 8, 745789. 34901061 10.3389/fmed.2021.745789 PMC8652056

[70] Studart-Neto A (2020) Neurological consultations and diagnoses in a large, dedicated COVID-19 university hospital. Arquivos de Neuro-Psiquiatria 78, 494–500. 32756734 10.1590/0004-282x20200089

[71] Iltaf Sr S (2020) Frequency of neurological presentations of coronavirus disease in patients presenting to a tertiary care hospital during the 2019 coronavirus disease pandemic. Cureus 12, 1–7. 10.7759/cureus.9846 PMC7497771 32953353

[72] Trigo J (2020) Factors associated with the presence of headache in hospitalized COVID-19 patients and impact on prognosis: a retrospective cohort study. The Journal of Headache and Pain 21, 1–10. 32727345 10.1186/s10194-020-01165-8 PMC7388434

[73] Ding Q (2021) Protein expression of angiotensin-converting enzyme 2 (ACE2) is upregulated in brains with Alzheimer's disease. International Journal of Molecular Sciences 22, 1687. 33567524 10.3390/ijms22041687 PMC7914443

[74] Quincozes-Santos A (2021) Association between molecular markers of COVID-19 and Alzheimer's disease. Journal of Medical Virology 94, 833–835. 34647635 10.1002/jmv.27391 PMC8662010

[75] Novak N (2021) SARS-CoV-2, COVID-19, skin and immunology–what do we know so far? Allergy 76, 698–713. 32658359 10.1111/all.14498 PMC7404682

[76] Farinazzo E (2021) Synthesis of the data on COVID-19 skin manifestations: underlying mechanisms and potential outcomes. Clinical, Cosmetic and Investigational Dermatology 14, 991. 34385830 10.2147/CCID.S325552 PMC8354337

[77] Colmenero I (2020) SARS-CoV-2 endothelial infection causes COVID-19 chilblains: histopathological, immunohistochemical and ultrastructural study of seven paediatric cases. British Journal of Dermatology 183, 729–737. 32562567 10.1111/bjd.19327 PMC7323219

[78] Cappel MA, Cappel JA and Wetter DA (2021) Pernio (chilblains), SARS-CoV-2, and COVID toes unified through cutaneous and systemic mechanisms. in mayo clinic proceedings. Elsevier 96, 989–1005. 10.1016/j.mayocp.2021.01.009 PMC7826004 33714595

[79] Assil S (2019) Plasmacytoid dendritic cells and infected cells form an interferogenic synapse required for antiviral responses. Cell Host & Microbe 25, 730–745, e6. 31003939 10.1016/j.chom.2019.03.005

[80] Lim K-H and Staudt LM (2013) Toll-like receptor signaling. Cold Spring Harbor Perspectives in Biology 5, a011247. 23284045 10.1101/cshperspect.a011247 PMC3579400

[81] Barrat FJ and Su L (2019) A pathogenic role of plasmacytoid dendritic cells in autoimmunity and chronic viral infection. Journal of Experimental Medicine 216, 1974–1985. 31420375 10.1084/jem.20181359 PMC6719431

[82] Maldonado MD, Romero-Aibar J and Pérez-San-Gregorio M (2021) COVID-19 pandemic as a risk factor for the reactivation of herpes viruses. Epidemiology & Infection 149, e145. 34130765 10.1017/S0950268821001333 PMC8245334

[83] Gallman AE and Fassett MS (2021) Cutaneous pathology of COVID-19 as a window into immunologic mechanisms of disease. Dermatologic Clinics 39, 533–543. 34556243 10.1016/j.det.2021.05.008 PMC8297957

[84] Herrada AA (2010) Aldosterone promotes autoimmune damage by enhancing Th17-mediated immunity. The Journal of Immunology 184, 191–202. 19949098 10.4049/jimmunol.0802886

[85] Magro C (2021) The skin as a critical window in unveiling the pathophysiologic principles of COVID-19. Clinics in Dermatology 39, 934–965. 34920833 10.1016/j.clindermatol.2021.07.001 PMC8298003

[86] Eriksson O (2020) Mannose-binding lectin is associated with thrombosis and coagulopathy in critically ill COVID-19 patients. Thrombosis and Haemostasis 120, 1720–1724. 32871607 10.1055/s-0040-1715835 PMC7869044

[87] Dang EV (2011) Control of TH17/Treg balance by hypoxia-inducible factor 1. Cell 146, 772–784. 21871655 10.1016/j.cell.2011.07.033 PMC3387678

[88] Jahani M, Dokaneheifard S and Mansouri K (2020) Hypoxia: a key feature of COVID-19 launching activation of HIF-1 and cytokine storm. Journal of Inflammation 17, 1–10. 33139969 10.1186/s12950-020-00263-3 PMC7594974

[89] Zuo Y (2020) Prothrombotic autoantibodies in serum from patients hospitalized with COVID-19. Science Translational Medicine 12, eabd3876. 33139519 10.1126/scitranslmed.abd3876 PMC7724273

[90] Consiglio CR (2020) The immunology of multisystem inflammatory syndrome in children with COVID-19. Cell 183, 968–981, e7. 32966765 10.1016/j.cell.2020.09.016 PMC7474869

[91] Di Minno A (2020) COVID-19 and venous thromboembolism: a meta-analysis of literature studies. in seminars in thrombosis and hemostasis. Thieme Medical Publishers 46, 763–771. 10.1055/s-0040-1715456 PMC7645842 32882719

[92] Klok F (2020) Incidence of thrombotic complications in critically ill ICU patients with COVID-19. Thrombosis Research 191, 145–147. 32291094 10.1016/j.thromres.2020.04.013 PMC7146714

[93] Levi M (2020) Coagulation abnormalities and thrombosis in patients with COVID-19. The Lancet Haematology 7, e438–e440. 32407672 10.1016/S2352-3026(20)30145-9 PMC7213964

[94] Shaw RJ (2021) COVID-19 and immunothrombosis: emerging understanding and clinical management. British Journal of Haematology 194, 518–529. 34114204 10.1111/bjh.17664

[95] Engelmann B and Massberg S (2013) Thrombosis as an intravascular effector of innate immunity. Nature Reviews Immunology 13, 34–45. 10.1038/nri3345 23222502

[96] Frantzeskaki F, Armaganidis A and Orfanos SE (2017) Immunothrombosis in acute respiratory distress syndrome: cross talks between inflammation and coagulation. Respiration; International Review of Thoracic Diseases 93, 212–225. 27997925 10.1159/000453002

[97] Ito T (2014) PAMPS and DAMPs as triggers for DIC. Journal of Intensive Care 2, 1–9. 25705424 10.1186/s40560-014-0065-0 PMC4336279

[98] Gould T, Lysov Z and Liaw P (2015) Extracellular DNA and histones: double-edged swords in immunothrombosis. Journal of Thrombosis and Haemostasis 13, S82–S91. 26149054 10.1111/jth.12977

[99] Alhamdi Y and Toh CH (2016) The role of extracellular histones in haematological disorders. British Journal of Haematology 173, 805–811. 27062156 10.1111/bjh.14077

[100] Veras FP (2020) SARS-CoV-2–triggered neutrophil extracellular traps mediate COVID-19 pathology. Journal of Experimental Medicine 217, e20201129. 32926098 10.1084/jem.20201129 PMC7488868

[101] Ng H (2021) Circulating markers of neutrophil extracellular traps are of prognostic value in patients with COVID-19. Arteriosclerosis, Thrombosis, and Vascular Biology 41, 988–994. 33267662 10.1161/ATVBAHA.120.315267 PMC7837697

[102] Schurink B (2020) Viral presence and immunopathology in patients with lethal COVID-19: a prospective autopsy cohort study. The Lancet Microbe 1, e290–e299. 33015653 10.1016/S2666-5247(20)30144-0 PMC7518879

[103] Goshua G (2020) Endotheliopathy in COVID-19-associated coagulopathy: evidence from a single-centre, cross-sectional study. The Lancet Haematology 7, e575–e582. 32619411 10.1016/S2352-3026(20)30216-7 PMC7326446

[104] Bryckaert M (2015) Of von Willebrand factor and platelets. Cellular and Molecular Life Sciences 72, 307–326. 25297919 10.1007/s00018-014-1743-8 PMC4284388

[105] Zheng XL (2015) ADAMTS13 and von Willebrand factor in thrombotic thrombocytopenic purpura. Annual Review of Medicine 66, 211–225. 10.1146/annurev-med-061813-013241 PMC4599565 25587650

[106] Stockschlaeder M, Schneppenheim R and Budde U (2014) Update on von Willebrand factor multimers: focus on high-molecular-weight multimers and their role in hemostasis. Blood Coagulation & Fibrinolysis 25, 206. 24448155 10.1097/MBC.0000000000000065 PMC3969155

[107] Katneni UK (2019) von Willebrand factor/ADAMTS-13 interactions at birth: implications for thrombosis in the neonatal period. Journal of Thrombosis and Haemostasis 17, 429–440. 30593735 10.1111/jth.14374

[108] Helms J (2020) High risk of thrombosis in patients with severe SARS-CoV-2 infection: a multicenter prospective cohort study. Intensive Care Medicine 46, 1089–1098. 32367170 10.1007/s00134-020-06062-x PMC7197634

[109] Lee SJ (2016) Thrombotic risk of reduced ADAMTS13 activity in patients with antiphospholipid antibodies. Blood Coagulation & Fibrinolysis 27, 907–912. 26757014 10.1097/MBC.0000000000000512

[110] Galeano-Valle F (2020) Antiphospholipid antibodies are not elevated in patients with severe COVID-19 pneumonia and venous thromboembolism. Thrombosis Research 192, 113–115. 32425261 10.1016/j.thromres.2020.05.017 PMC7227496

[111] Bowles L (2020) Lupus anticoagulant and abnormal coagulation tests in patients with COVID-19. New England Journal of Medicine 383, 288–290. 32369280 10.1056/NEJMc2013656 PMC7217555

[112] Austin S (2008) The VWF/ADAMTS13 axis in the antiphospholipid syndrome: ADAMTS13 antibodies and ADAMTS13 dysfunction. British Journal of Haematology 141, 536–544. 18341632 10.1111/j.1365-2141.2008.07074.x

[113] Katneni UK (2020) Coagulopathy and thrombosis as a result of severe COVID-19 infection: a microvascular focus. Thrombosis and Haemostasis 120, 1668–1679. 32838472 10.1055/s-0040-1715841 PMC7869056

[114] Bazzan M (2020) Low ADAMTS 13 plasma levels are predictors of mortality in COVID-19 patients. Internal and Emergency Medicine 15, 861–863. 32557383 10.1007/s11739-020-02394-0 PMC7300200

[115] Marco A and Marco P (2021) Von Willebrand factor and ADAMTS13 activity as clinical severity markers in patients with COVID-19. Journal of Thrombosis and Thrombolysis 52, 497–503. 33866481 10.1007/s11239-021-02457-9 PMC8053027

[116] Dolhnikoff M (2020) Pathological evidence of pulmonary thrombotic phenomena in severe COVID-19. Journal of Thrombosis and Haemostasis: JTH 18, 1517–1519. 32294295 10.1111/jth.14844 PMC7262093

[117] Fletcher-Sandersjöö A and Bellander B-M (2020) Is COVID-19 associated thrombosis caused by overactivation of the complement cascade? A literature review. Thrombosis Research 194, 36–41. 32569879 10.1016/j.thromres.2020.06.027 PMC7301826

[118] Testa S (2020) Direct oral anticoagulant plasma levels’ striking increase in severe COVID-19 respiratory syndrome patients treated with antiviral agents: the Cremona experience. Journal of Thrombosis and Haemostasis 18, 1320–1323. 32329231 10.1111/jth.14871 PMC7264501

[119] Schutgens RE (2021) DOAC in COVID-19: yes or no? HemaSphere 5, e526. 33403357 10.1097/HS9.0000000000000526 PMC7773328

